# DNA ploidy of primary breast cancer and local recurrence after breast-conserving therapy.

**DOI:** 10.1038/bjc.1991.257

**Published:** 1991-07

**Authors:** H. Beerman, B. A. Bonsing, M. J. van de Vijver, J. Hermans, P. M. Kluin, R. J. Caspers, C. J. van de Velde, C. J. Cornelisse

**Affiliations:** Department of Pathology, Faculty of Medicine, Leiden University Hospital, University of Leiden, The Netherlands.

## Abstract

The value of DNA-flow cytometry and clinico-pathological prognostic factors for the prediction of local recurrences after breast-conserving therapy (BCT) were evaluated in a retrospective study. Thirty-one patients with a local recurrence were compared with 31 matched patients without a local recurrence. Morphology and DNA-indices of the local recurrences and their corresponding primary tumours were compared. Ductal carcinoma in situ was present significantly more often in the group with a primary recurring tumour, than in the matched group (P less than 0.001), and the same holds for lobular carcinoma (n = 5). Half of the tumours that recurred had macroscopically positive surgical margins compared to about one-fourth of the matched group. Fifty-six per cent of the DNA-aneuploid stemlines in cases with local recurrence were present in the corresponding primary tumour as well (confidence limits 45%-75%), an indication that the majority of local recurrences are true recurrences and not independently developed tumours. The lack of similarity of DNA stemlines between some primary DNA-aneuploid tumours and their local recurrences indicates that these tumours had developed independently. The percentage of DNA-aneuploid cases in the group with local recurrence (89%) did not differ significantly from that in the matched group (70%). However, the findings suggest a selective recurrence of DNA-diploid stemlines. This might indicate increased resistance of DNA-diploid tumour cells to radiotherapy as compared with the resistance level in DNA-aneuploid cells.


*Br. J. Cancer* (1991), **64**, 139–143 © Macmillan Press Ltd., 1991

### DNA ploidy of primary breast cancer and local recurrence after breast-conserving therapy

H. Beerman[1], B.A. Bonsing[1], M.J. van de Vijver[1], J. Hermans[2], Ph.M. Kluin[1], R.J. Caspers[3], C.J.H. van de Velde[4] & C.J. Cornelisse[1]

*Departments of [1]Pathology, [2]Medical Statistics, [3]Radiotherapy and [4]Surgery, Faculty of Medicine and Leiden University Hospital, University of Leiden, Leiden, The Netherlands.*

**Summary** The value of DNA-flow cytometry and clinico-pathological prognostic factors for the prediction of local recurrences after breast-conserving therapy (BCT) were evaluated in a retrospective study. Thirty-one patients with a local recurrence were compared with 31 matched patients without a local recurrence. Morphology and DNA-indices of the local recurrences and their corresponding primary tumours were compared. Ductal carcinoma *in situ* was present significantly more often in the group with a primary recurring tumour, than in the matched group ($P < 0.001$), and the same holds for lobular carcinoma ($n = 5$). Half of the tumours that recurred had macroscopically positive surgical margins compared to about one-fourth of the matched group. Fifty-six per cent of the DNA-aneuploid stemlines in cases with local recurrence were present in the corresponding primary tumour as well (confidence limits 45%–75%), an indication that the majority of local recurrences are true recurrences and not independently developed tumours. The lack of similarity of DNA stemlines between some primary DNA-aneuploid tumours and their local recurrences indicates that these tumours had developed independently. The percentage of DNA-aneuploid cases in the group with local recurrence (89%) did not differ significantly from that in the matched group (70%). However, the findings suggest a selective recurrence of DNA-diploid stemlines. This might indicate increased resistance of DNA-diploid tumour cells to radiotherapy as compared with the resistance level in DNA-aneuploid cells.

Breast-conserving therapy (BCT) is being applied increasingly in the primary treatment of early breast cancer (Eberlein *et al.*, 1990). One of the main concerns here is the continued risk of local recurrence in the retained portion of a breast. Unlike local recurrences after radical mastectomy, which usually coincide with a systemic disease, most local recurrences after BCT can be adequately treated (Barr *et al.*, 1989; Cady, 1990). This means that follow up of patients treated by BCT and identification of women at high risk of local recurrence development are essential. The question as to whether a local recurrence itself constitutes a risk of developing distant metastases remains to be answered. The published reports indicate that a local recurrence does not significantly influence overall survival, but the number of follow-up years is still small (Calle *et al.*, 1986; Fisher *et al.*, 1989).

A major goal for the improvement of BCT is the identification of patients, who are at increased risk of developing a local recurrence. Important risk factors are the presence of a large component of DCIS in the primary tumour (Dongen van *et al.*, 1989) and non-radical surgical margins (Kurtz *et al.*, 1990*a*). Whether the DNA content can contribute to prediction of local recurrences has been the subject of only one investigation (Cooke *et al.*, 1988). In that study use was made of DNA image cytometry and a positive association was found between abnormal DNA content distribution and the risk of local recurrence. In general, DNA-aneuploidy determined by flow cytometry has been found in many studies to be associated with a poorer prognosis for breast cancer (Clark *et al.*, 1989; Beerman *et al.*, 1990). In the present retrospective study we therefore assessed the value of DNA-flow cytometry for the identification of patients at high risk of local recurrence after BCT. Our results indicate that DNA-flow cytometry does not promote this identification of individual patients at high risk of local recurrence. Comparison of primary tumours and their local recurrences often showed similarity in DNA-aneuploid stemlines, indicating that a least 50% of local recurrences are true recurrences and not independently developed tumours. Furthermore, our results suggest a decreased sensitivity of DNA-diploid tumour cells to radiotherapy.

Correspondence: H. Beerman, Department of Pathology, Dr Danielden Hoed Kliniek, PO Box 5201, 3008AE, Rotterdam, The Netherlands.
Received 19 October 1990; and in revised form 1 February 1991.

### Materials and methods

### *Patients*

During the period between January 1980 and December 1988, 251 patients with primary breast carcinoma $<3$ cm received similar treatment consisting of lumpectomy and irradiation in the Leiden University Hospital (AZL), Leiden (The Netherlands). Up to December 1989, 26 (10.4%) of these patients had developed a local recurrence. For five patients who had been treated at an affiliated general hospital in Leiden and who developed a local recurrence, surgical records, histological records, slides, and paraffin-embedded material were available and were put at the disposal of the Department of Pathology of the Leiden University Hospital. All of the patients had received post-operative radiotherapy of 50 Gy on the breast and a 15 Gy boost by electrons on the tumour bed in the Department of Radiotherapy of the Leiden University Hospital. The interval to local recurrence was calculated as starting at the time of the primary surgical treatment. Each patient with a local recurrence was matched with another patient without a local recurrence, who had a follow-up period similar to that of the disease-free period of recurrent primary tumours. For 27 recurrent primary tumours, 27 matched tumours, and 25 local recurrences, enough tumour material was available to allow flow cytometric analysis. The two cases of recurrence for which neither histological examination nor flow cytometry was performed, were diagnosed on the basis of the information supplied by fine-needle aspiration. Patients were not matched for age. A complete chronology of each patient's disease history including data on initial treatment and follow-up was retrieved from the Surgical Documenation Service (AZL). Patients were staged according to the post-surgical TNM classification (UICC). The average follow-up period, the patient's age (at the time of diagnosis), TNM stage, tumour size, and nodal status are listed in Table I.

*Histological classification*

Histological sections of all of the tumours were reviewed independently by two of the present authors (HB and MV). The results of the macroscopical examination of surgical margins were retrieved from the reports of the Department of Pathology. For this retrospective investigation, inked microscopical surgical margins were only available for a limited number of cases and were not further evaluated.

Infiltrating ductal carcinomas with <75% DCIS were graded according to a modified Bloom and Richardson classification (Elston, 1987). With this method tubule formation and nuclear pleomorphism are assessed and the mitotic rate is determined. For all cases the fractional area of tumour tissue of DCIS was independently estimated by two of the authors (HB, MV) by visual examination of all available histological slides. In case of a disagreement (>10%), the slides were re-examined until agreement was reached. On this basis the infiltrating ductal carcinomas were assigned to one of five categories according to the percentage of DCIS: 0%, 1–25%, 26–75%, 76–99%, and 100%. The DCIS were classified according to their growth pattern as a comedo and a non-comedo type and, according to their nuclear pleomorphism, as small or large type. This classification is simple and seems to have a biological basis (van de Vijver *et al.*, 1988; Baird *et al.*, 1990). Infiltrating ductal carcinomas with >25% DCIS in the tumour were called ductal carcinomas with an extensive intraductal component. Infiltrating lobular carcinomas were not subdivided.

For histodiagnostic purposes, multiple tissue blocks were routinely taken from different parts of the primary tumour specimen and stored after being paraffin-embedded or frozen at −70°C. For DNA flow cytometry, we retrieved all available tissue blocks from the archives of the Department of Pathology.

**Table I** Clinico-pathological variables and histological data on 31 primary tumours and 31 matched control tumours

| | *Primary tumours n = 31* Mean | s.d. | *Matched tumours n = 31* Mean | s.d. | $P^a$ |
|---|---|---|---|---|---|
| Follow-up time (months) | 34.4 | (21.8) | 35.7 | (21.4) | |
| Age (years) | 46.7 | (11.6) | 47.2 | (11.6) | N.S. |
| Tumour size (mm) | 23.1 | (11.9) | 21.0 | ( 7.7) | N.S. |
| Stage | | | | | N.S. |
| 0 | 6 | | 0 | | |
| II | 8 | | 14 | | |
| IIA | 9 | | 12 | | |
| IIB | 5 | | 2 | | |
| Unknown | 3 | | 3 | | |
| Tumour size | | | | | N.S. |
| Tx | 3 | | 3 | | |
| Tis | 6 | | 0 | | |
| T1 | 12 | | 14 | | |
| T2 | 9 | | 14 | | |
| T3 | 1 | | 0 | | |
| Nodal status | | | | | N.S. |
| N0 | 23 | | 27 | | |
| N1 | 8 | | 4 | | |
| Histology | | | | | |
| Lobular carcinoma | 5 | | 0 | | – |
| Ductal carcinoma | 26 | | 31 | | – |
| Grade I | 2 | | 7 | | N.S. |
| Grade II | 9 | | 18 | | N.S. |
| Grade III | 4 | | 3 | | N.S. |
| Percentage DCIS | | | | | |
| 0 | 1 | | 20 | | <0.01 |
| <0– ⩽25 | 5 | | 6 | | N.S. |
| <25– ⩽100 | 14 | | 5 | | <0.01 |
| 100 | 6 | | 0 | | <0.05 |
| Comedo type DCIS | 19 | | 7 | | <0.01 |

[a]McNemar test applied to paired data. [b]Only ductal carcinomas with <75% DCIS were graded (*n* = 15).

*DNA flow cytometry*

The cell preparation and staining procedures used for fresh and paraffin-embedded tissue have been described elsewhere (Cornelisse *et al.*, 1987). Briefly, suspensions of isolated nuclei were prepared from fresh or frozen tissue specimens according to Vindelov's detergent-trypsin procedure and were stained with propidium iodide (Vindelov *et al.*, 1983). Samples of deparaffinised tissue were stained with propidium iodide. The DNA content was determined with an FACscan flow cytometry (Becton Dickinson, San Jose, CA, USA) with the appropriate filter combinations for the excitation and measurements of PI. On average, about 10,000 cells per sample were measured. Histological sections of frozen-tissue and/or paraffin sections were cut from each tissue block to permit estimation of the percentage of tumour cells, before and after sectioning for flow cytometric analysis. The percentage of tumour cells was estimated visually by two independent observers (HB and CJC). If the estimated percentages differed by more than 10%, the slides were re-examined in a joint session until agreement was reached. All slides showing less than 10%, or more than 90% tumour cells were considered unsuitable for flow cytometric analysis in paraffin-embedded material. Rainbow trout red blood cells were added to the suspensions of isolated nuclei prepared from the frozen samples as an internal ploidy standard. For deparaffinised samples this was not possible, and here non-neoplastic cells in the tumour specimen (e.g. stroma cells and lymphocytes) served for internal ploidy control. The pepsin-digestion technique was used to release nuclei from 45 μm sections of paraffin-embedded tissue specimens (Hedley *et al.*, 1983).

The average number of analysed samples of recurrent primary tumours, matched tumours, and local recurrences is 4.7 (s.d. = 2.0), 3.7 (s.d. = 1.1), and 3.7 (s.d. = 1.0), respectively. These numbers are fairly close to the minimal number of four samples found in a previous study to be required to adequately establish intra-tumour DNA-stemline heterogeneity (Beerman *et al.*, 1991).

*Classification of DNA-ploidy status*

DNA profiles showing only one $G_{0,1}$ cell population, with a CV of <5.5%, were classified as diploid, and those with one or more additional $G_{0,1}$ peaks as DNA-aneuploid. For frozen samples, the position of the DNA-diploid peak was verified with the use of the TRBC standard. In DNA profiles obtained in deparaffinised samples showing two or more distinct $G_{0,1}$ populations, the one at the extreme left was considered to represent the non-neoplastic DNA-diploid cell population. Tumours showing a single $G_{0,1}$ peak with a CV >5.5% were classified as 'wide CV DNA-diploid'. These were pooled with the other DNA-diploid tumours, whereas $G_{0,1}$ peaks with CV >10% were considered non-evaluable. tumour ploidy was expressed as the DNA index (Hiddemann *et al.*, 1984). The average CV for paraffin-embedded material amounted to 5.5 (s.d. = 1.8, range 2.5–9.6) and for frozen tumour samples to 3.0 (s.d. = 1.0, range 2.0–6.4).

Tumours showing two or more different DNA-aneuploid stemlines, in the same or different samples, were classified as multiploid. We considered two DNA-aneuploid stemlines to be different, if both gave distinct $G_{0,1}$ peaks in the same DNA profile, or the DNA indices of different tumour samples differed by more than 10%. The term intra-tumour heterogeneity covers not only multiploidy but also tumours having a single DNA-diploid stemline as well as a DNA-aneuploid stemline in two separate tumour samples. A DNA-diploid $G_{0,1}$ peak accompanied by a DNA-aneuploid $G_{0,1}$ peak in the same DNA profile was not considered to represent a tumour DNA-stemline. A DNA-diploid tumour cell population was identified by the presence of a single $G_{0,1}$ peak in DNA profiles of specimens with an estimated tumour cellularity greater than 20%.

*Statistical analysis*

Statistical analysis was performed with the BMDP and SSPS statistical packages. The frequency distribution of DNA-

ploidy stemlines in recurrent primary tumours, matched tumours, and local recurrences was compared by the Kolmogorov-Smirnov test. The differences between recurrent primary tumours, matched tumours, and/or local recurrences in relation to clinicopathological variables and flow cytometric results were analysed by the McNemar test on paired data. $P$ values $<0.05$ were considered significant.

### Results

### *Comparison of recurrent primary tumours and matched tumours*

*Clinico-pathological variables* Several morphological differences were found between tumours with and without a local recurrence. In almost all (25/26) ductal-type recurrent primary tumours, a component of ductal carcinoma *in situ* was present. This frequency is significantly higher than that found for the matched tumours (11/26) ($P<0.01$) (Table I). There were also significantly more ductal carcinomas with more than 25% DCIS in recurrent primary tumours than in matched tumours (14/26 *vs* 5/26; $P<0.01$). In a paired comparison, DCIS of the comedo type were found to occur significantly more frequently in tumours that recurred than in the matched tumours. Among tumours the five locally recurring primary tumours were diagnosed as infiltrating lobular carcinoma, whereas none of the matched tumours was lobular. Tumours with local recurrences showing positive macroscopical surgical margins more often than matched tumours (16/31 *vs* 7/31) did. Age, nodal status, TNM stage, tumour size, and histological grade did not differ significantly between the two groups (Table I).

### *DNA-ploidy*

DNA flow cytometry applied to 27 tumours that recurred locally and the tumours in the 27 matched patients, showed a high degree of intra-tumour DNA stemline heterogeneity for both recurrent primary and matched tumours. The average number of distinct tumour DNA-stemlines, including both DNA-aneuploid and DNA-diploid tumour stemlines, was similar for recurrent primary tumours ($n = 2.1$, s.d. = 1.0, range 1–5) and matched tumours ($n = 1.8$, s.d. = 0.7, range 1–3). Of the recurrent primary tumours, 89% (24/27) showed DNA-aneuploidy *vs* 70% (19/27) of the matched tumours, but the paired comparison showed that these differences were not statistically significant. There were no significant differences in the percentage of either low DNA-aneuploid (DNA-index $<1.40$) or high DNA-aneuploid (DNA-index $\geqslant 1.40$) stemlines (Beerman *et al.*, 1990) (Table II).

### *Comparison of recurrent primary tumours and local recurrences*

*Clinico-pathological variables* Recurrence-free intervals of infiltrating lobular carcinoma and infiltrating ductal carcinoma did not differ significantly (43 months, s.d. = 16.4, range 11–59 *vs* 34 months, s.d. = 22.4, range 4–88). The two small cell type DCIS recurred much later than the four large cell type DCIS did (average 68.5 months *vs* 38.5 months). Of the six recurrent primary tumours composed solely of DCIS, only two were DCIS after recurrence, the other four showed less than 25% DCIS in the presence of infiltrating ductal carcinoma. In five cases of recurrent primary tumours with $>75\%$ DCIS, four of the local recurrences showed only infiltrating carcinoma, the others more than 75% DCIS.

*DNA-ploidy* The average number of distinct tumour DNA-stemlines in local recurrences (1.5, s.d. = 0.6, range 1–3) was significantly lower than that in the primary tumours of the same patients ($P = 0.01$) (Table III). In 56% (9/16) of the local recurrences with a DNA-aneuploid stemline the same DNA-stemline could be traced in the primary tumour (confidence limits 45%–75%). In a high percentage (18/25) of the local recurrences a DNA-diploid tumour cell population was identified and half of this group was purely DNA-diploid (in the other nine cases also a distinct DNA-aneuploid stemline was also found in a separate sample of the same tumour). In the corresponding nine primary tumours only one was purely DNA-diploid; in the other eight primary tumours (six had both DNA-diploid and DNA-aneuploid stemlines and two only DNA-aneuploid stemlines) (Table III).

Comparisons between the percentages of low aneuploid (DNA-index $<1.40$) and high aneuploid tumours (DNA-index $\geqslant 1.40$) in primary tumours and their local recurrences showed no statistically significantly differences.

The average recurrence-free interval for patients in whom the primary tumour and local recurrence showed one or more identical aneuploid DNA-stemlines was not significantly shorter, than for those without such DNA-stemline similarity (33.9 months, s.d. = 20.7, range 4–76 *vs* 49.4 months, s.d. = 25.1, range 17–88).

### Discussion

Our results confirm an association between the presence of an intraductal component, particularly of the comedo type, and an increased risk of local recurrence. These findings are in agreement with most of the reports in the literature (Lind-

**Table II** DNA ploidy in primary tumours (PT), matched control tumours (MT), and local recurrences (LR)

| | *PT* $n = 27$ | *MC*[a] $n = 27$ | *LR*[a] $n = 25$ |
|---|---|---|---|
| DNA-diploid | 3 | 8 | 9 |
| DNA-aneuploid | 24 | 19 | 16 |
| DNA-diploid + DNA-aneuploid[b] | 15 | 14 | 18 |
| DNA-index $>1.40$ | 18 | 16 | 11 |
| Multiploidy | 10 | 11 | 4 |

[a]Paired comparison (McNemar) shows no statistically significant differences between PT/MT and PT/LR. [b]DNA-diploid and DNA-aneuploid tumour stemlines in separate samples of the same tumour.

**Table III** Comparative data on DNA stemlines in 25 primary tumours and 25 local recurrences. For all cases, indentical DNA stemlines are only presented once

| *Local recurrence* | | | *Primary tumour* | | | | |
|---|---|---|---|---|---|---|---|
| DNA-diploid ($n = 9$) | | | | | | | |
| 1.0 | | | 1.0 | | | | |
| 1.0 | | | 1.0 | 1.1 | | | |
| 1.0 | | | 1.0 | 1.1 | | | |
| 1.0 | | | 1.0 | 1.8 | | | |
| 1.0 | | | 1.0 | 1.9 | | | |
| 1.0 | | | 1.0 | 2.2 | | | |
| 1.0 | | | 1.0 | 1.1 | 1.2 | | |
| 1.0 | | | 1.6 | 1.7 | 1.8 | 2.1 | |
| 1.0 | | | 1.7 | | | | |
| Presence of similar DNA-aneuploid stemlines ($n = 9$) | | | | | | | |
| 1.0 | 1.1 | | 1.0 | 1.1 | 1.3 | | |
| 1.0 | 1.2 | | 1.0 | 1.2 | | | |
| 1.0 | 1.7 | | 1.7 | | | | |
| 1.0 | 1.9 | | 1.9 | | | | |
| 1.1 | 1.8 | 2.1 | 1.0 | 1.3 | 2.1 | | |
| 1.2 | 1.6 | | 1.2 | 1.6 | 1.8 | | |
| 1.3 | 2.1 | | 1.1 | 1.3 | 1.9 | 2.1 | 3.2 |
| 1.5 | | | 1.1 | 1.5 | | | |
| 2.1 | | | 1.0 | 1.3 | 1.9 | 2.1 | 2.5 |
| Absence of similar DNA-aneuploid stemlines ($n = 7$) | | | | | | | |
| 1.0 | 1.5 | | 1.0 | 1.2 | | | |
| 1.0 | 1.6 | | 1.0 | 1.9 | | | |
| 1.0 | 1.1 | | 1.0 | | | | |
| 1.0 | 1.3 | | 1.0 | | | | |
| 1.0 | 1.1 | | 1.5 | 1.7 | | | |
| 1.2 | | | 1.8 | 2.1 | | | |
| 2.2 | | | 1.9 | | | | |

ley *et al.*, 1989; Baird *et al.*, 1990; Locker *et al.*, 1990). The tendency of DCIS to be multifocal or multicentric in about 25–50% of the cases has been thought to be responsible for the high local recurrence rate (Schwartz *et al.*, 1980; Holland *et al.*, 1990). Furthermore, the shorter local recurrence-free period in patients with a high-grade DCIS *vs* those with a well-differentiated DCIS seen in the present study has already been reported (Lagios *et al.*, 1989). A distinctly high incidence of infiltrating lobular carcinoma in the group with a local recurrence may indicate a higher risk of local recurrence for this group. However, consensus has not been reached in the literature on this subject (Mate *et al.*, 1986; du Toit *et al.*, 1989; Schnitt *et al.*, 1989; van Limbergen *et al.*, 1990). All five of these cases of recurrent lobular carcinoma in our study showed a remarkable similarity of the histological picture. These tumours were composed of multiple lobular carcinoma foci separated from each other by non-neoplastic breast parenchyme, indicating a possible multifocality or multicentricity. A higher local recurrence risk of this tumour type might be accounted for by its often diffuse growth pattern and lack of dominant or well-defined mass.

In the present study the local recurrence rate was not significantly associated with age, tumour size, TNM stage, histological grade, or nodal status. The small size of the tumours in our series may have obscured such relationships; even in larger-scale studies these correlations are still controversial (Harris *et al.*, 1985; Fisher *et al.*, 1986; Lindley *et al.*, 1989; Locker *et al.*, 1989; Kurtz *et al.*, 1990*b*). In cases that did recur, the tumour specimens had macroscopically positive margins more often than those in the matched control group did. Similar observations have been made in other studies (Kurtz *et al.*, 1990*a*).

The similarity between DNA-aneuploid stemlines in 56% of local recurrences and primary tumours supports the opinion that these local recurrences are true recurrences and not independently developing tumours. The likelihood that two independent tumours will have the same aneuploid DNA-indices has been shown by a recent statistical analysis to be very small (Smit *et al.*, 1990). Sofar, the evidence indicating true local recurrence has been based solely on the similarity in histology and location in or adjacent to the initial tumour bed (Clarke *et al.*, 1985; Fisher *et al.*, 1986). However, in six cases no concordance was found for DNA-aneuploid stemlines between local recurrences and primary tumours, even after extensive sampling of both the recurrence and the primary tumour. In these cases an independent, multicentric origin of the primary tumour and local recurrence is feasible.

Although not statistically significant, our data suggest that there may be some kind of association between DNA-aneuploidy and an increased recurrence rate. This is in line with the results presented to the EORTC *In Situ* Breast Cancer Workshop in 1988 (Cooke *et al.*, 1988). However, this relationship is complicated by our finding that among 22 patients with DNA-aneuploid recurrent primary tumours there was a relatively high percentage (36) of the recurrences showing a purely DNA-diploid stemline. In six cases where primary tumours showed both DNA-aneuploid and DNA-diploid tumour stemlines, only the DNA-diploid stemlines recurred. This might indicate that DNA-diploid tumour cell populations are more resistant to radiotherapy than DNA-aneuploid populations are.

The high frequency of DNA-aneuploid stemlines in the present series is similar to the 88% found in a DNA image cytometry study of 26 cases of DCIS + micro-invasive carcinoma (Carpenter *et al.*, 1987). Generally, the reported percentage of DNA-aneuploidy in the literature is lower (Kallioniemi *et al.*, 1987; Clark *et al.*, 1989). This divergence might be due to undersampling, since in the present study (with extensive sampling) the calculated probability that a single sample will be DNA-aneuploidy was only 64.9%, which is well within the range reported in the literature. Similar results for DNA-aneuploidy were obtained in a recent flow-cytometric study on tumour heterogeneity (Beerman *et al.*, 1991). Furthermore, the frequent coexistence of DNA-diploid and DNA-aneuploid tumour cell populations in our series of tumours might be attributable to the presence of a DCIS component in most of the recent primary tumours. DCIS are more frequently DNA-diploid, especially the non-comedo type, than infiltrating carcinomas are (Carpenter *et al.*, 1987; Locker *et al.*, 1990).

In sum, the present results confirm the conclusion that there is higher risk of local recurrence for tumours with an extensive intra-ductal component, and also indicate a higher risk for infiltrating lobular carcinomas. The findings also support the importance of macroscopically free surgical margins, and do not definite proof that DNA-aneuploidy constitutes a major risk factor for local recurrence after BCT. The higher incidence of DNA-diploid stemlines among cases of local recurrence raises important questions about the sensitivity of DNA-diploid tumour cell populations to radiotherapy. Moreover, the concordance in DNA-aneuploidy between primary tumours and local recurrences in more than 50% of the cases supports the notion that a substantial proportion of local recurrences can be attributed to the non-radical excision of tumour tissue.

### References

BAIRD, R.M., WORTH, A. & HISLOP, G. (1990). Recurrence after lumpectomy for comedo-type intraductal carcinoma of the breast. *Am. J. Surg.*, **159**, 479.

BARR, L.C., BRUNT, A.M., GOODMAN, A.G., PHILLIPS, R.H. & ELLIS, H. (1989). Uncontrolled local recurrence after treatment of breast cancer with breast conservation. *Cancer*, **64**, 1203.

BEERMAN, H., KLUIN, Ph.M., HERMANS, J., VAN DE VELDE, C.J.H. & CORNELISSE, C.J. (1990). Prognostic significance of DNA-ploidy in a series of 690 primary breast cancer patients. *Int. J. Cancer*, **45**, 34.

BEERMAN, H., SMIT, V.T.H.B.M., KLUIN, Ph.M., BONSING, B.A., HERMANS, J. & CORNELISSE, C.J. (1991). Flow cytometric analysis of DNA stemline heterogeneity in primary and metastatic breast cancer. *Cytometry*, **12**, 147.

CADY, B. (1990). New diagnostic, staging, and therapeutic aspects of early breast cancer. *Cancer*, **65**, 634.

CALLE, R., VILCOQ, J.R., ZAFRANI, B., VIELH, P. & FOURQUET, A. (1986). Local control and survival of breast cancer treated by limited surgery followed by irradiation. *Int. J. Radiat. Oncol. Biol. Phys.*, **12**, 873.

CARPENTER, R., GIBBS, N., MATTHEWS, J. & COOKE, T. (1987). Importance of cellular DNA content in pre-malignant breast disease and pre-invasive carcinoma of the female breast. *Br. J. Surg.*, **74**, 905.

CLARK, G.M., DRESSLER, L.G., OWENS, M.A., POUNDS, G., OLDAKER, T. & MCGUIRE, W.L. (1989). Prediction of relapse or survival in patients with node-negative breast cancer by DNA flow cytometry. *N. Engl. J. Med.*, **320**, 627.

CLARKE, D.H., LÊ, M.G., SARRAZIN, D. & 6 others (1985). Analysis of local-regional relapses in patients with early breast cancers treated by excision and radiotherapy: experience at the Institut Gustave-Roussy. *Int. J. Radiat. Oncol. Biol. Phys.*, **11**, 137.

COOKE, T. & CARPENTER, R. (1988). Prognostic value of cellular DNA content in the management of ductal carcinoma *in situ*. EORTC, *In situ* Breast Cancer Workshop, 13. (Abstract)

CORNELISSE, C.J., VAN DE VELDE, C.J.H., CASPERS, R.J., MOOLENAAR, A.J. & HERMANS, J. (1987). DNA ploidy and survival in breast cancer patients. *Cytometry*, **8**, 225.

DONGEN VAN, J.A., FENTIMAN, I.S., HARRIS, J.R. & 4 others (1989). *In situ* breast cancer: the EORTC consensus meeting. *Lancet*, 125.

DU TOIT, R.S., LOCKER, A.P., ELLIS, I.O., ELSTON, C.W., NICHOLSON, R.I. & BLAMEY, R.W. (1989). Invasive lobular carcinomas of the breast-the prognosis of histopathological subtypes. *Br. J. Cancer*, **60**, 605.

EBERLEIN, T.J., CONNOLLY, J.L., SCHNITT, S.J., RECHT, A., OSTEEN, R.T. & HARRIS, J.R. (1990). Predictors of local recurrence following conservative breast surgery and radiation therapy. *Arch. Surg.*, **125**, 771.

ELSTON, C.W. (1987). Grading of invasive carcinoma of the breast. In: *Diagnostic Pathology of the Breast*, Page, D.L. & Anderson, D.W. (eds). New York: Churchill Livingstone, pp. 300-311.

FISHER, B., REDMOND, C., POISSON, R. & 12 others (1989). Eight-years results of a randomized clinical trial comparing total mastectomy and lumpectomy with or without irradiation in the treatment of breast cancer. *N. Eng. J. Med.*, **320**, 822.

FISHER, E.R., SASS, R., FISHER, B., GREGORIO, R., BROWN, R. & WICKERHAM, L. (1986). Pathologic findings from the national surgical adjuvant breast project (Protocol 6). II. Relation of local breast recurrence to multicentricity. *Cancer*, **57**, 1717.

HARRIS, J.R., CONNOLLY, J.L., SCHNITT, S.J. & 8 others (1985). The use of pathological features in selecting the extent of surgical resection necessary for breast cancer patients treated by primary radiation therapy. *Ann. Surg.*, **201**, 164.

HEDLEY, D.W., FRIEDLANDER, M.L., TAYLOR, I.W., RUGG, C.A. & MUSGROVE, E.A. (1983). Method for analysis of cellular DNA content of paraffin-embedded pathologic material using flow cytometry. *J. Histochem. Cytochem.*, **31**, 1333.

HIDDEMANN, W., SCHUMANN, J., ANDREEFF, M. & 6 others (1984). Convention on nomenclature for DNA cytometry. *Cytometry*, **5**, 445.

HOLLAND, R., CONNOLLY, J.L., GELMAN, R. & 7 others (1990). The presence of an extensive intraductal component following a limited excision correlates with prominent residual disease in the remainder of the breast. *J. Clin. Oncol.*, **8**, 113.

KALLIONIEMI, O.P., BLANCO, G., ALAVAIKKO, M. & 4 others (1987). Tumour DNA ploidy as an independent prognostic factor in breast cancer. *Br. J. Cancer*, **56**, 637.

KURTZ, J.M., JACQUEMIER, J., AMALRIC, R. & 6 others (1990*a*). Risk factors for breast recurrence in premenopausal and postmenopausal patients with ductal cancers treated by conservation therapy. *Cancer*, **65**, 1867.

KURTZ, J.M., JACQUEMIER, J., AMALRIC, R. & 5 others (1990*b*). Why are local recurrences after breast conserving therapy more frequent in younger patients. *J. Clin. Oncol.*, **8**, 591.

LAGIOS, M.D., MARGOLIN, F.R., WESTDAHL, P.R. & ROSE, M.R. (1989). Mammographically detected duct carcinoma *in situ*. Frequency of local recurrence following tylectomy and prognostic effect of nuclear grade on local recurrence. *Cancer*, **63**, 618.

VAN LIMBERGEN, E., VAN DER SCHUEREN, E., VAN DEN BOGAERT, W. & VAN WING, J. (1990). Local control of operable breast cancer after radiotherapy alone. *Eur. J. Cancer*, **26**, 674.

LINDLEY, R., BULMAN, A., PARSONS, P., PHILLIPS, R., HENRY, K. & ELLIS, H. (1989). Histologic features predictive of an increased risk of early local recurrence after treatment of breast cancer by local tumor excision and radical radiotherapy. *Surgery*, **105**, 13.

LOCKER, A.P., HORROCKS, C., GILMOUR, A.S. & others (1990). Flow cytometric and histological analysis of ductal carcinoma *in situ* of the breast. *Br. J. Surg.*, **77**, 564.

MATE, T.P., CARTER, D., FISCHER, D.B. & 4 others (1986). A clinical and histopathological analysis of the results of conservation surgery and radiation therapy in Stage I and II breast carcinomas. *Cancer*, **58**, 1995.

SCHNITT, S.J., CONNOLLY, J.L., RECHT, A., SILVER, B. & HARRIS, J.R. (1989). Influence of infiltrating lobular histology on local tumor control in breast cancer patients treated with conservative surgery and radiotherapy. *Cancer*, **64**, 448.

SCHWARTZ, G.F., PATCHEFSKY, A.S., FEIG, A., SHABER, G.S. & SCHWARTZ, A.B. (1980). Multicentricity and implications for treatment. *Ann. Surg.*, **191**, 8.

SMIT, V.T.H.B.M., FLEUREN, G.J., VAN HOUWELINGEN, J.C., ZEGVELD, S., KUIPERS-DIJKSHOORN, N.J. & CORNELISSE, C.J. (1990). Flow cytometric DNA-ploidy analysis indicates a metastatic origin of most multiple malignancies of the female tract. *Cancer*, **66**, 1843.

VAN DE VIJVER, M.J., PETERSE, J.L., MOOI, W.J. & 4 others (1988). Neu-protein overexpression in breast cancer: association with comedo-type ductal carcinoma *in situ* and limited prognostic value in stage II breast cancer. *N. Eng. J. Med.*, **319**, 1239.

VINDELOV, L.L., CHRISTENSEN, I.J., JENSEN, G. & NISSEN, N.I. (1983). Limits of detection of nuclear DNA abnormalities by flow cytometric DNA analysis. *Cytometry*, **3**, 332.


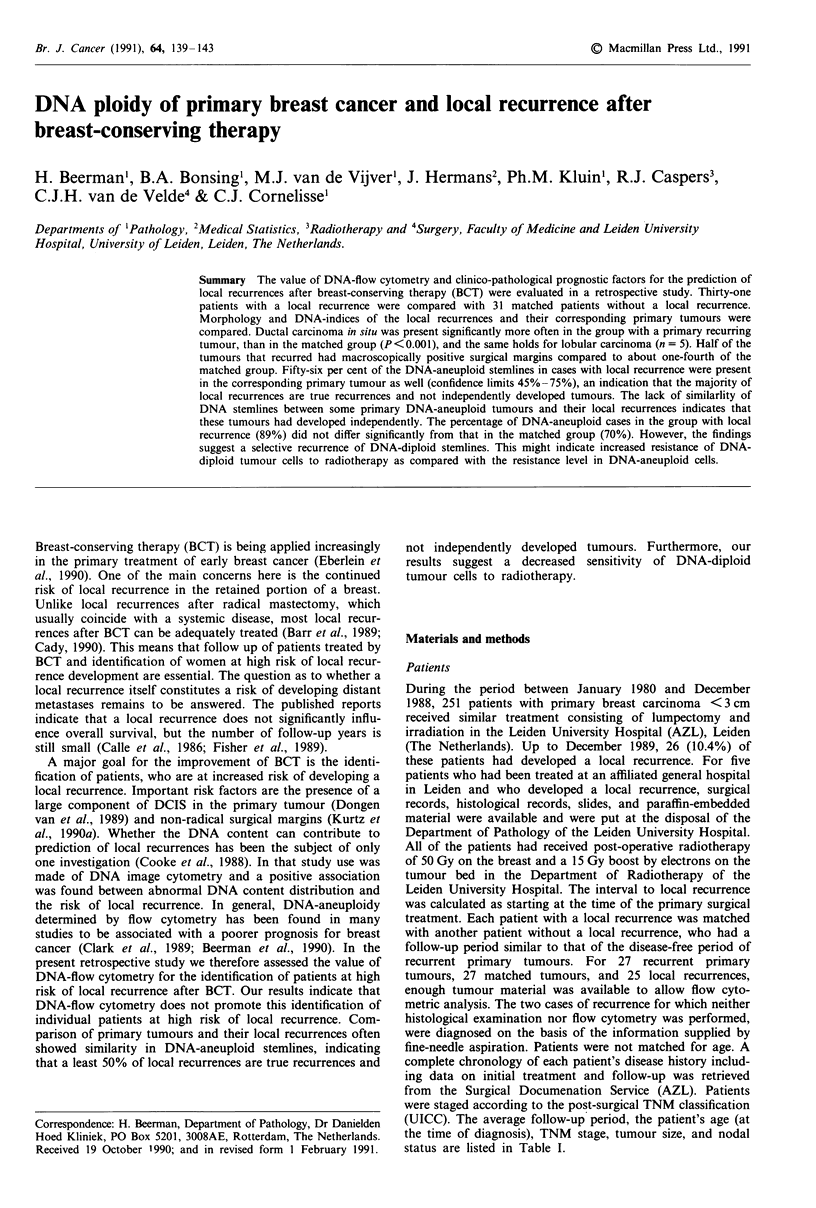

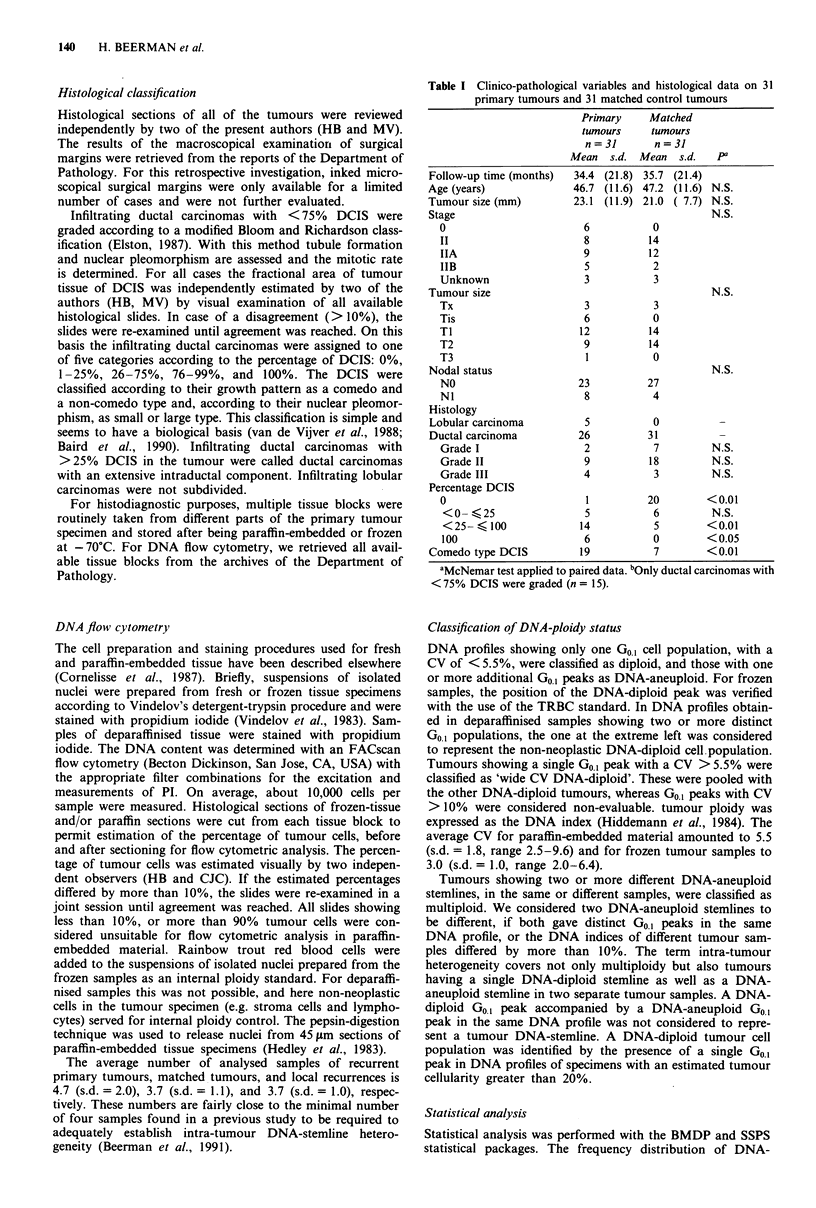

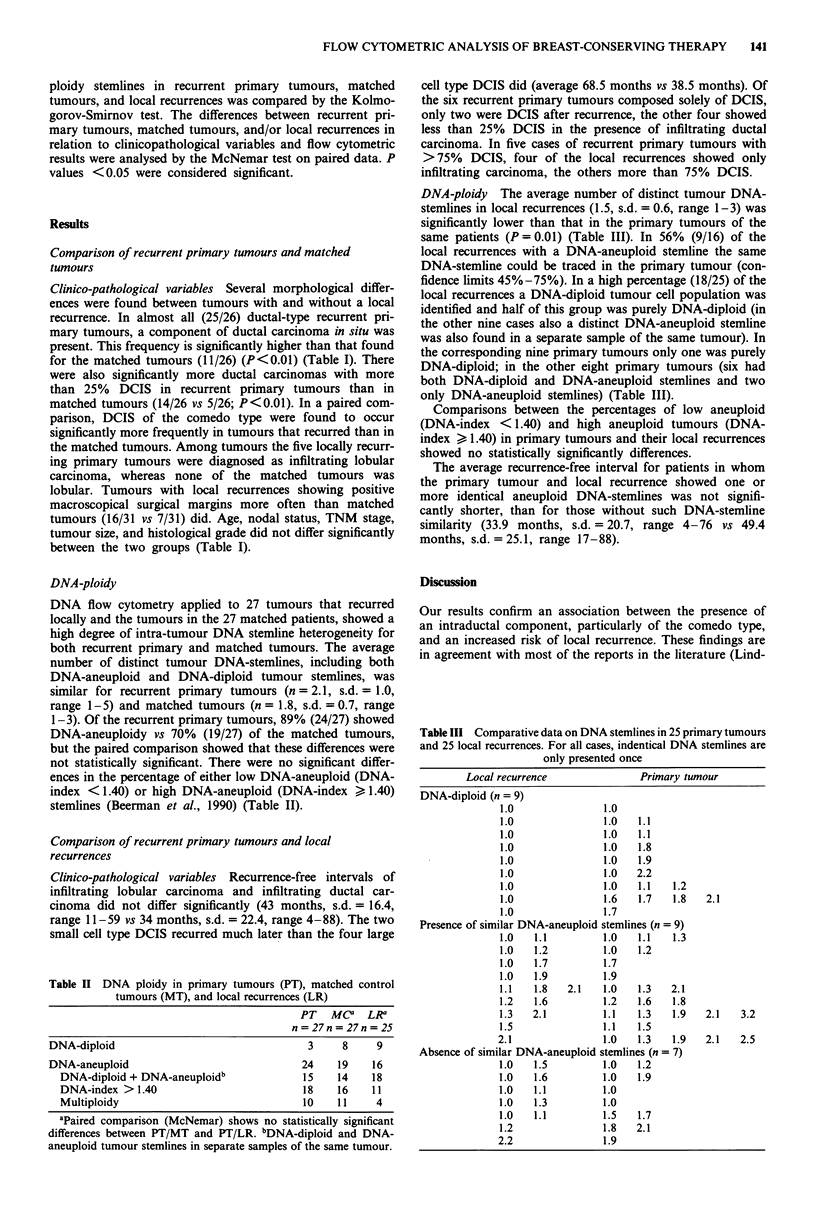

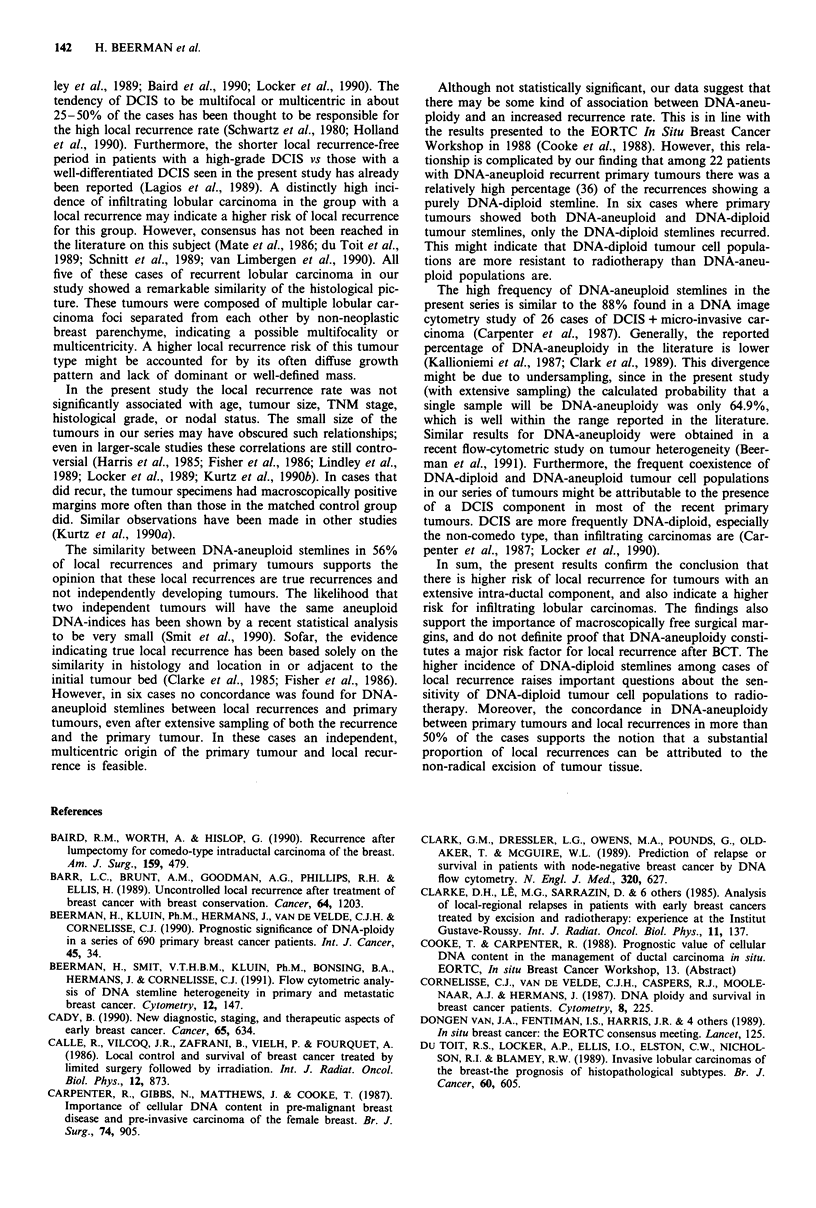

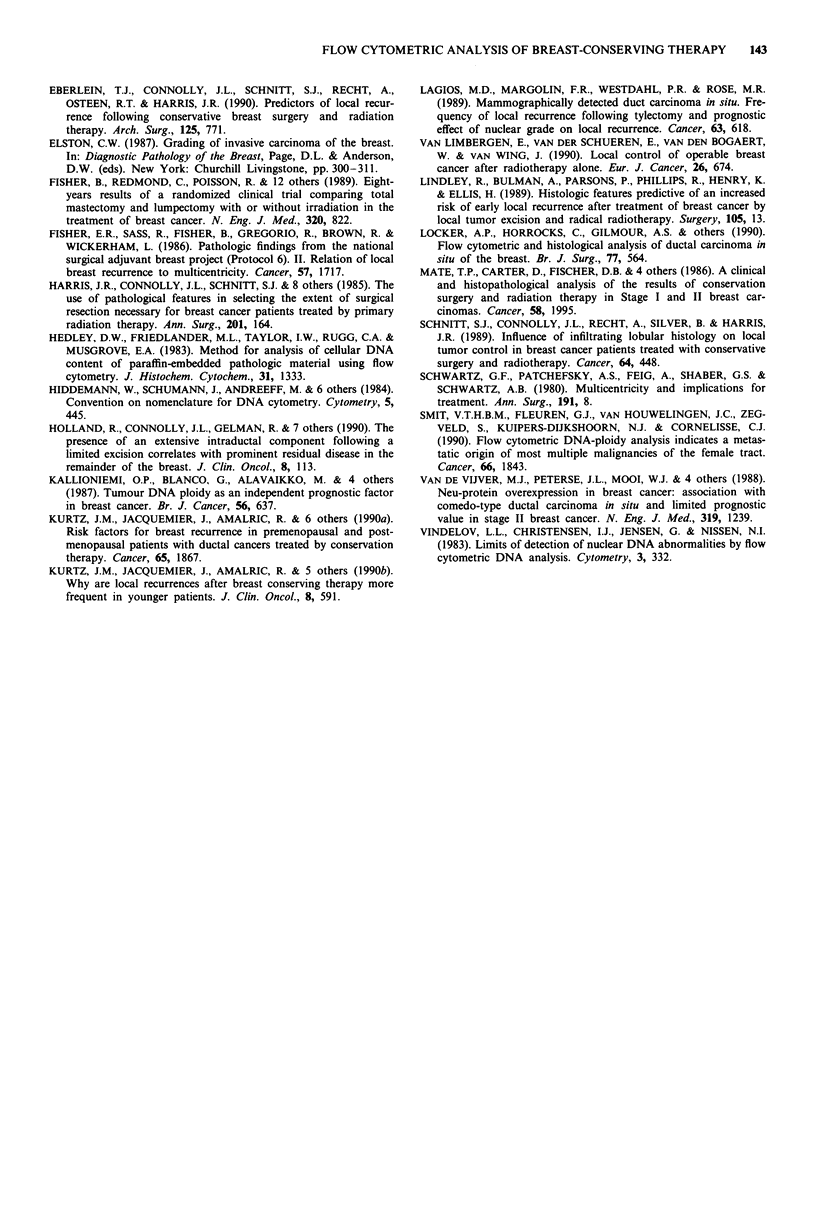


## References

[OCR_00569] Baird R. M., Worth A., Hislop G. (1990). Recurrence after lumpectomy for comedo-type intraductal carcinoma of the breast.. Am J Surg.

[OCR_00574] Barr L. C., Brunt A. M., Goodman A. G., Phillips R. H., Ellis H. (1989). Uncontrolled local recurrence after treatment of breast cancer with breast conservation.. Cancer.

[OCR_00581] Beerman H., Kluin P. M., Hermans J., van de Velde C. J., Cornelisse C. J. (1990). Prognostic significance of DNA-ploidy in a series of 690 primary breast cancer patients.. Int J Cancer.

[OCR_00587] Beerman H., Smit V. T., Kluin P. M., Bonsing B. A., Hermans J., Cornelisse C. J. (1991). Flow cytometric analysis of DNA stemline heterogeneity in primary and metastatic breast cancer.. Cytometry.

[OCR_00591] Cady B. (1990). New diagnostic, staging, and therapeutic aspects of early breast cancer.. Cancer.

[OCR_00595] Calle R., Vilcoq J. R., Zafrani B., Vielh P., Fourquet A. (1986). Local control and survival of breast cancer treated by limited surgery followed by irradiation.. Int J Radiat Oncol Biol Phys.

[OCR_00601] Carpenter R., Gibbs N., Matthews J., Cooke T. (1987). Importance of cellular DNA content in pre-malignant breast disease and pre-invasive carcinoma of the female breast.. Br J Surg.

[OCR_00609] Clark G. M., Dressler L. G., Owens M. A., Pounds G., Oldaker T., McGuire W. L. (1989). Prediction of relapse or survival in patients with node-negative breast cancer by DNA flow cytometry.. N Engl J Med.

[OCR_00613] Clarke D. H., Lê M. G., Sarrazin D., Lacombe M. J., Fontaine F., Travagli J. P., May-Levin F., Contesso G., Arriagada R. (1985). Analysis of local-regional relapses in patients with early breast cancers treated by excision and radiotherapy: experience of the Institut Gustave-Roussy.. Int J Radiat Oncol Biol Phys.

[OCR_00626] Cornelisse C. J., van de Velde C. J., Caspers R. J., Moolenaar A. J., Hermans J. (1987). DNA ploidy and survival in breast cancer patients.. Cytometry.

[OCR_00640] Eberlein T. J., Connolly J. L., Schnitt S. J., Recht A., Osteen R. T., Harris J. R. (1990). Predictors of local recurrence following conservative breast surgery and radiation therapy. The influence of tumor size.. Arch Surg.

[OCR_00651] Fisher B., Redmond C., Poisson R., Margolese R., Wolmark N., Wickerham L., Fisher E., Deutsch M., Caplan R., Pilch Y. (1989). Eight-year results of a randomized clinical trial comparing total mastectomy and lumpectomy with or without irradiation in the treatment of breast cancer.. N Engl J Med.

[OCR_00657] Fisher E. R., Sass R., Fisher B., Gregorio R., Brown R., Wickerham L. (1986). Pathologic findings from the National Surgical Adjuvant Breast Project (protocol 6). II. Relation of local breast recurrence to multicentricity.. Cancer.

[OCR_00663] Harris J. R., Connolly J. L., Schnitt S. J., Cady B., Love S., Osteen R. T., Patterson W. B., Shirley R., Hellman S., Cohen R. B. (1985). The use of pathologic features in selecting the extent of surgical resection necessary for breast cancer patients treated by primary radiation therapy.. Ann Surg.

[OCR_00669] Hedley D. W., Friedlander M. L., Taylor I. W., Rugg C. A., Musgrove E. A. (1983). Method for analysis of cellular DNA content of paraffin-embedded pathological material using flow cytometry.. J Histochem Cytochem.

[OCR_00682] Holland R., Connolly J. L., Gelman R., Mravunac M., Hendriks J. H., Verbeek A. L., Schnitt S. J., Silver B., Boyages J., Harris J. R. (1990). The presence of an extensive intraductal component following a limited excision correlates with prominent residual disease in the remainder of the breast.. J Clin Oncol.

[OCR_00686] Kallioniemi O. P., Blanco G., Alavaikko M., Hietanen T., Mattila J., Lauslahti K., Koivula T. (1987). Tumour DNA ploidy as an independent prognostic factor in breast cancer.. Br J Cancer.

[OCR_00691] Kurtz J. M., Jacquemier J., Amalric R., Brandone H., Ayme Y., Hans D., Bressac C., Roth J., Spitalier J. M. (1990). Risk factors for breast recurrence in premenopausal and postmenopausal patients with ductal cancers treated by conservation therapy.. Cancer.

[OCR_00697] Kurtz J. M., Jacquemier J., Amalric R., Brandone H., Ayme Y., Hans D., Bressac C., Spitalier J. M. (1990). Why are local recurrences after breast-conserving therapy more frequent in younger patients?. J Clin Oncol.

[OCR_00702] Lagios M. D., Margolin F. R., Westdahl P. R., Rose M. R. (1989). Mammographically detected duct carcinoma in situ. Frequency of local recurrence following tylectomy and prognostic effect of nuclear grade on local recurrence.. Cancer.

[OCR_00713] Lindley R., Bulman A., Parsons P., Phillips R., Henry K., Ellis H. (1989). Histologic features predictive of an increased risk of early local recurrence after treatment of breast cancer by local tumor excision and radical radiotherapy.. Surgery.

[OCR_00718] Locker A. P., Horrocks C., Gilmour A. S., Ellis I. O., Dowle C. S., Elston C. W., Blamey R. W. (1990). Flow cytometric and histological analysis of ductal carcinoma in situ of the breast.. Br J Surg.

[OCR_00723] Mate T. P., Carter D., Fischer D. B., Hartman P. V., McKhann C., Merino M., Prosnitz L. R., Weissberg J. B. (1986). A clinical and histopathologic analysis of the results of conservation surgery and radiation therapy in stage I and II breast carcinoma.. Cancer.

[OCR_00729] Schnitt S. J., Connolly J. L., Recht A., Silver B., Harris J. R. (1989). Influence of infiltrating lobular histology on local tumor control in breast cancer patients treated with conservative surgery and radiotherapy.. Cancer.

[OCR_00735] Schwartz G. F., Patchefsky A. S., Feig S. A., Shaber G. S., Schwartz A. B. (1980). Clinically occult breast cancer. Multicentricity and implications for treatment.. Ann Surg.

[OCR_00742] Smit V. T., Fleuren G. J., van Houwelingen J. C., Zegveld S. T., Kuipers-Dijkshoorn N. J., Cornelisse C. J. (1990). Flow cytometric DNA-ploidy analysis of synchronously occurring multiple malignant tumors of the female genital tract.. Cancer.

[OCR_00708] Van Limbergen E., Van der Schueren E., Van den Bogaert W., Van Wing J. (1990). Local control of operable breast cancer after radiotherapy alone.. Eur J Cancer.

[OCR_00753] Vindeløv L. L., Christensen I. J., Jensen G., Nissen N. I. (1983). Limits of detection of nuclear DNA abnormalities by flow cytometric DNA analysis. Results obtained by a set of methods for sample-storage, staining and internal standardization.. Cytometry.

[OCR_00634] du Toit R. S., Locker A. P., Ellis I. O., Elston C. W., Nicholson R. I., Blamey R. W. (1989). Invasive lobular carcinomas of the breast--the prognosis of histopathological subtypes.. Br J Cancer.

[OCR_00747] van de Vijver M. J., Peterse J. L., Mooi W. J., Wisman P., Lomans J., Dalesio O., Nusse R. (1988). Neu-protein overexpression in breast cancer. Association with comedo-type ductal carcinoma in situ and limited prognostic value in stage II breast cancer.. N Engl J Med.

